# Antiretroviral therapy partially improves the abnormalities of dendritic cells and lymphoid and myeloid regulatory populations in recently infected HIV patients

**DOI:** 10.1038/s41598-019-48185-2

**Published:** 2019-08-12

**Authors:** Mercedes Márquez-Coello, Montserrat Montes de Oca Arjona, Andrés Martín-Aspas, Francisca Guerrero Sánchez, Clotilde Fernández-Gutiérrez del Álamo, José A. Girón-González

**Affiliations:** 1 Unidad de Enfermedades Infecciosas, Servicio de Medicina Interna, Hospital Universitario Puerta del Mar, Facultad de Medicina, Universidad de Cádiz, Cádiz, Spain; 2 Instituto de Investigación e Innovación en Ciencias Biomédicas de la Provincia de Cádiz (INiBICA), Cádiz, Spain; 3 Servicio de Microbiología, Hospital Universitario Puerta del Mar, Facultad de Medicina, Universidad de Cádiz, Cádiz, Spain

**Keywords:** Diseases, HIV infections

## Abstract

This study aimed to evaluate the effects of antiretroviral therapy on plasmacytoid (pDC) and myeloid (mDC) dendritic cells as well as regulatory T (Treg) and myeloid-derived suppressor (MDSC) cells in HIV-infected patients. Forty-five HIV-infected patients (20 of them with detectable HIV load −10 recently infected and 10 chronically infected patients-, at baseline and after antiretroviral therapy, and 25 with undetectable viral loads) and 20 healthy controls were studied. The influence of HIV load, bacterial translocation (measured by 16S rDNA and lipopolysaccharide-binding protein) and immune activation markers (interleukin –IL- 6, soluble CD14, activated T cells) was analyzed. The absolute numbers and percentages of pDC and mDC were significantly increased in patients. Patients with detectable viral load exhibited increased intracellular expression of IL-12 by mDCs and interferon -IFN- α by pDCs. Activated population markers were elevated, and the proportion of Tregs was significantly higher in HIV-infected patients. The MDSC percentage was similar in patients and controls, but the intracellular expression of IL-10 was significantly higher in patients. The achievement of undetectable HIV load after therapy did not modify bacterial translocation parameters, but induce an increase in pDCs, mDCs and MDSCs only in recently infected patients. Our data support the importance of early antiretroviral therapy to preserve dendritic and regulatory cell function in HIV-infected individuals.

## Introduction

Viral and bacterial infections are initially recognized by specialized antigen-presenting cells, including dendritic cells (DCs), which can be classified into two groups: plasmacytoid dendritic cells (pDCs) and myeloid dendritic cells (mDCs). The pDC response is mediated through the recognition of viral genomic RNA by Toll-like receptor (TLR) 7 or through the recognition of double-stranded DNA by TLR9. pDCs secrete interferon alpha (IFN-α), which induces an antiviral state and promotes the activation of natural cytotoxic cells^1^. Recognition by mDCs is mediated by TLRs expressed in the cellular membrane (TLR1, TLR2, TLR4, TLR5, TLR6, TLR10 and TLR11) or in the interior of the cell (TLR3 and TLR8). mDCs secrete interleukin (IL)-12 and promote an immune T helper 1 (Th1) cell response^2^.

The number of pDCs increases during the acute phase of human immunodeficiency virus (HIV) infection, when circulating IFN-α is detected at higher levels, and declines in patients with chronic HIV infection^3–5^. The pDC depletion has been attributed to apoptosis^6^ or to migration of pDCs towards gut-associated lymphoid tissues and lymph nodes^7^. Although mDCs are not significantly reduced in subjects with acute HIV infection^8^, a decrease is observed during the chronic phase^9^, mainly in those with a lower CD4 T cell count^10^. These data are opposite to the anticipated strong innate immune response after the recognition of viral antigens in other diseases^1,2^.

Immune activation is present in HIV-infected patients, even in those in which viral replication is controlled and CD4+ T cells are normal^11^. In fact, immune activation markers are considered a prognostic index in HIV-infected individuals^11^. Among others, the drivers of immune activation include microbial translocation phenomena as a result of intestinal barrier damage^11–13^. After acute infection, a massive depletion of CD4+ T cells from gut-associated lymphoid tissues occurs and persists during the chronic stage^14,15^. The administration of antiretroviral therapy (ART) only partially repairs gut mucosal injury^11,12^.

The consequences of bacterial translocation, secondary to intestinal barrier damage, on innate and adaptive immune function have been insufficiently analyzed in HIV-infected patients. Thus, the impact of bacterial translocation on DCs has received limited attention. Even though bacterial lipopolysaccharide (LPS) is recognized by mDCs via TLR4, the expected increased secretion of IL-12 is usually abnormal in HIV infection. Also, the expected IFN-α secretion after recognition via TLR9 of the bacterial DNA by pDCs is also defective in these patients^16^.

The regulatory mechanisms of persistent immune activation include regulatory T lymphocytes (Tregs) and myeloid-derived suppressor cells (MDSCs). Tregs suppress T lymphocyte proliferation and DCs function through contact mechanisms and by the secretion of cytokines (transforming growth factor beta 1 (TGF-β1) or IL-10)^17^. We previously reported that the proportion of Tregs is increased in HIV-infected patients with uncontrolled HIV replication^18^.

MDSCs are a heterogeneous population comprising myeloid progenitors and immature macrophages, granulocytes and dendritic cells. In humans, MDSCs represent approximately 0.5% of peripheral white blood cells. The exposure of precursors (peripheral blood or bone marrow mononuclear cells) to LPS or to proinflammatory cytokines contributes to the expansion of MDSCs^19^. Two types of human MDSC have been characterized. Both types express CD11b. Monocytic MDSC (M-MDSC) are characterized by the expression of CD14, and granulocytic polymorphonuclear MDSC (G-MDSC) by the expression of CD15 and CD66b. MDSCs induce the expansion of Tregs and block the DCs production by bone marrow^20^. An increase in G-MDSCs^21^ and M-MDSCs^22^ has been detected in HIV-infected patients, although the ability to secrete IL-10 or the relationship with bacterial translocation markers has not been analyzed.

We hypothesized that the changes in number or proportions of immune cells detected at baseline would be due to stimuli provided by HIV itself and by microbial translocation-derived products. After controlling HIV replication by ART, modulation of immune populations would be only the effect of these microbial translocation-derived molecules.

The objectives of the present work were: (1) To perform a quantitative evaluation of antigen-presenting and regulatory cells in untreated HIV-infected patients with recent or chronic infection. (2) To analyze the modifications of these parameters after ART. (3) To investigate the influence on those of HIV load, bacterial translocation and immune activation markers. (4) To analyze the persistence of the immune changes measuring these parameters in patients with undetectable HIV load after being treated for a long period.

## Results

The immune and virological characteristics of the untreated patients and controls are shown in Table 1.

**Table 1:** Immune and virological characteristics, bacterial translocation and monocyte and lymphocyte activation of healthy controls and HIV-infected patients.

	Healthy controls (n = 20)	Naïve patients with detectable HIV load (n = 20).		
		Overall (n = 20)	HIV infection, recent (n = 10)	HIV infection, chronic (n = 10)
Age (years)	43 (29–47)	37 (30–46)	32 (25–36)	44 (38–49)
Sex male	17 (85)	17 (85)	9 (90)	8 (80)
T CD4+ cells/mm^3^, at baseline	740 (629–1068)	387 (183–583)***	572 (449–824)*	199 (132–340)*** ^‡‡‡^
T CD8+ cells/mm^3^, at baseline	426 (374–530)	784 (431–1264)*	894 (679–1374)***	441 (265–1201)
HIV load (copies/ml) at baseline		24379 (2655–105980)	18142 (1440–37483)	63302 (4561–192592)
Presence of bacterial DNA in peripheral blood (n, %)	0 (0)	20 (100)***	10 (100)***	10 (100)***
LBP (ng/ml)	5·2 (4·3–5·6)	6·4 (5·3–9·9)*	6·2 (5·0–10·0)*	6·6 (5·7–10·4)**
IL-6 (pg/ml)	1·8 (1·3–1·9)	5·4 (4·6–8·6)***	5·7 (4·7–9·0)***	5·1 (4·3–7·9)***
sCD14 (ng/ml)	2325 (1879–2894)	3017 (2386–3582)**	2918 (2299–3470)*	3017 (2418–3615)*
T CD4+ DR+ cells (% of CD4+ lymphocytes)	7 (6–8)	19 (14–32)***	15 (13–17)**	32 (23–38)*** ^‡^
T CD8+ DR+ cells (% of CD8+ lymphocytes)	7 (5–9)	20 (11–21)***	14 (8–20)*	21 (18–36)*** ^‡^

Data are provided as absolute number (percentage) or as median (interquartile range).

HIV-infected patients vs healthy controls: *p < 0·05, **p < 0·01, ***p < 0·001.

Patients with recent infection vs patients with chronic infection: ^‡^p < 0·05, ^‡‡^p < 0·01, ^‡‡‡^p < 0·001.

### Bacterial translocation and monocyte and lymphocyte activation

16S rDNA was detected in peripheral blood of all patients. LBP was significantly higher in patients than in healthy controls. HIV-positive subjects exhibited a significantly higher serum concentration of IL-6 and sCD14 and higher percentages of CD4+ DR+ and CD8+ DR+ than healthy controls. The highest values of activated lymphocytes were observed in chronic HIV-infected patients with uncontrolled HIV replication (Table 1). A significant negative correlation was detected between baseline CD4+ T cell counts and LBP concentration (r = −0·440, p = 0·046).

### Dendritic cells in untreated HIV-infected patients and quantitation of the Th1 and Th2 subpopulations

**Figure 1:**
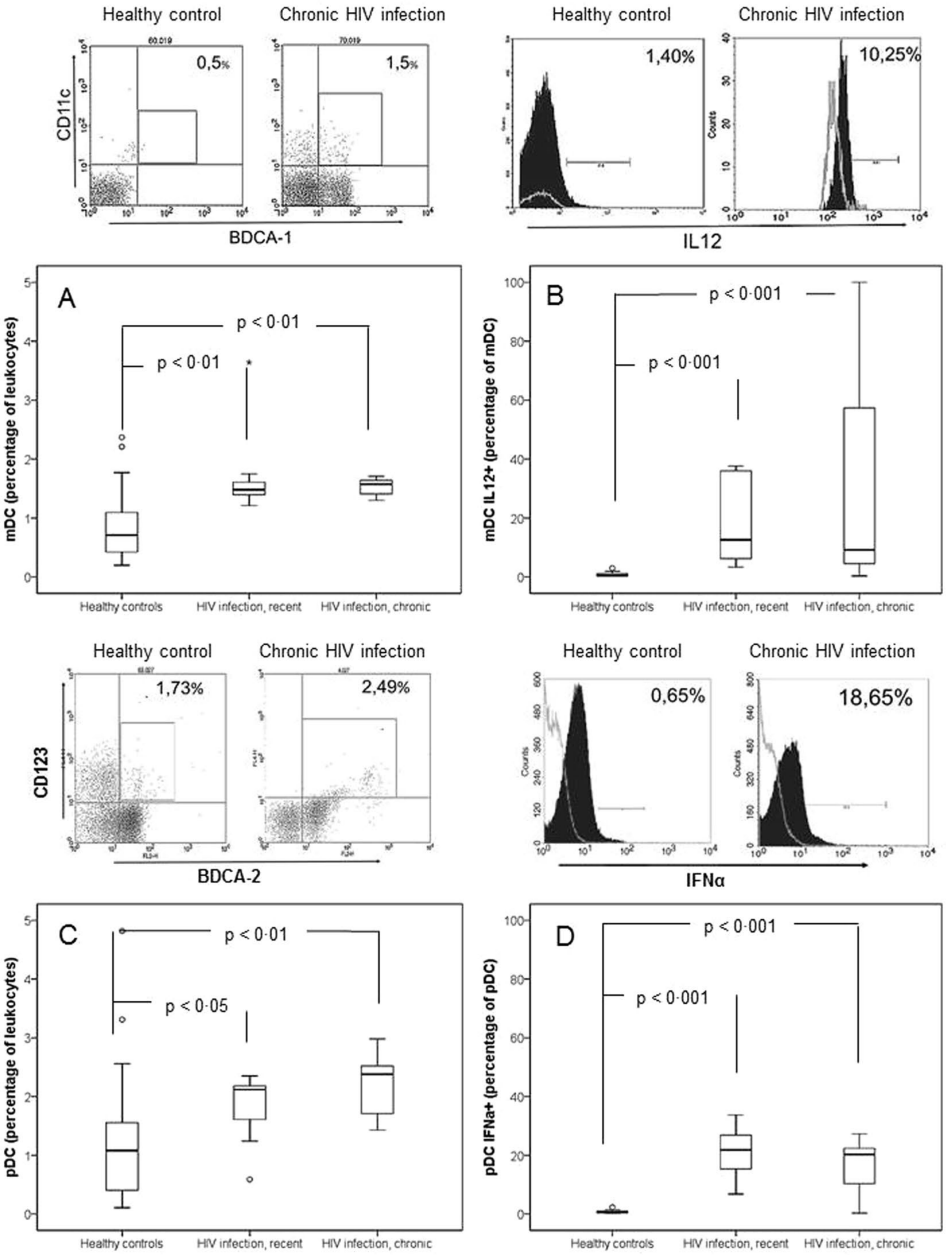
Proportions of dendritic cells and intracellular expression of cytokines by them in healthy controls (n = 20) and in untreated patients with HIV infection: recent (n = 10) and chronic (n = 10). (**A**) Myeloid dendritic cells –mDCs- (percentage of leukocytes). mDCs were identified by their reactivity with anti-CD1c (BDCA-1, clone AD5-8E7) (Miltenyi Biotec GMBH, Bergisch Gladbach, Germany) after exclusion of monocytes, B lymphocytes and dead cells using a cocktail of anti-CD14/anti-CD19/dead cells (Cocktail Lin clones CD3-SK3, CD14-Mφ9, CD16-3G8, CD19-SJ25L1, CD20-L27, CD56-NCAM16.2; BD Biosciences, Franklin Lakes, NJ, USA). (**B**) Intracellular expression of interleukin 12 (percentage of myeloid dendritic cells expressing interleukin 12), determined using specific antibodies (anti-IL12p40/70, clone C8.6, Miltenyi Biotec GMBH), after stimulation with LPS (1 μg/ml) for 5 hours. (**C**) Plasmocytoid dendritic cells –pDCs- (percentage of leukocytes). pDCs were identified by their reactivity with anti-CD303 (BDCA-2, clone AC-144) (Miltenyi Biotec GMBH, Bergisch Gladbach, Germany) after exclusion of monocytes, B lymphocytes and dead cells using a cocktail of anti-CD14/anti-CD19/dead cells (Cocktail Lin clones CD3-SK3, CD14-Mφ9, CD16-3G8, CD19-SJ25L1, CD20-L27, CD56-NCAM16.2; BD Biosciences, Franklin Lakes, NJ, USA). (**D**) Intracellular expression of interferon alpha (percentage of plasmacytoid dendritic cells expressing interferon alpha), determined using specific antibodies (clone LT 27:295, Miltenyi Biotec GMBH), after stimulation with CpG-A (3 μM) for 20 hours. Above each graphic, flow cytometry data for representative cases of a healthy control and a patient with untreated chronic HIV infection are shown. A grey line in (**B**,**C**) figures represents isotype controls, used to confirm specificity of staining and to discriminate background staining. Data are provided as median, interquartile values and range.

The absolute numbers and percentages of mDCs and pDCs were significantly higher in untreated HIV-infected individuals than in healthy donors. The percentage of mDC expressing IL-12 and pDC expressing IFN-α were significantly higher in untreated HIV individuals than in healthy controls (Table 2, Fig. 1). We did not observe significant differences in the absolute number or in the percentages of either type of DCs among the two groups of HIV-infected subjects (Table 2, Fig. 1). No significant correlation was detected between mDC or pDC and baseline CD4+ T lymphocytes or HIV load (data not shown).

**Table 2:** Number, percentage and expression of cytokines by dendritic cells of healthy controls and untreated HIV-infected patients.

	Healthy controls (n = 20)	Naïve patients with detectable HIV load (n = 20).		
		Overall (n = 20)	HIV infection, recent (n = 10)	HIV infection, chronic (n = 10)
mDC/mm^3^	39 (22–57)	81 (64–114)***	101 (70–173)**	79 (61–90)**
mDC (percentage of leukocytes)	0·7 (0.4–1.2)	1·6 (1·4–1·6)***	1·5 (1·3–1·7)**	1·6 (1·4–1·7)**
mDC expressing IL-12 (percentage of mDC)	0·6 (0·3–1·2)	12·5 (5·2–81·3)***	12·5 (5·2–50·0)***	11·9 (3·5–97·7)***
pDC/mm^3^	59 (21–108)	95 (80–169)*	94 (62–195)*	97 (80–137)*
pDC (percentage of leukocytes)	1·1 (0·4–1·6)	2·1 (1·7–2·4)***	2·1 (1·3–2·3)*	2·4 (1·7–2·6)**
pDC expressing IFN-α (percentage of pDC)	0·7 (0·3–1·1)	21·7 (14·2–26·0)***	22·6 (15·4–28·5)***	20·3 (9·6–22·9)***

Abbreviations: mDC: Myeloid dendritic cells; pDC: Plasmocytoid dendritic cells; IL-12: interleukin 12; IFN-α: interpheron alpha.

Data are provided as absolute number (percentage) or as median (interquartile range).

HIV-infected patients vs healthy controls: *p < 0·05, **p < 0·01, ***p < 0·001.

Patients with recent vs chronic infection: ^‡^p < 0·05, ^‡‡^p < 0·01, ^‡‡‡^p < 0·001.

Because DCs influence the adaptive immune response, we next analyzed the Th1 and Th2 subpopulations. HIV-infected individuals exhibit a significantly higher percentage of Th2 cells [healthy controls, 22 (14–26); patients with recent infection, 58 (24–69); patients with chronic infection, 63 (38–79); p < 0·05 with reference to healthy control in both cases. Data expressed as percentage of T CD4+ lymphocytes] without significant differences between the two groups of patients (p > 0·05). By contrast, Th1 cells number was similar in patients and healthy controls [healthy controls, 17 (11–25); patients with recent infection, 29 (18–51); patients with chronic infection, 38 (8–59); p > 0·05 with reference to healthy control in both cases. Data expressed as percentage of T CD4+ lymphocytes].

### Regulatory cells in HIV-infected patients

**Figure 2:**
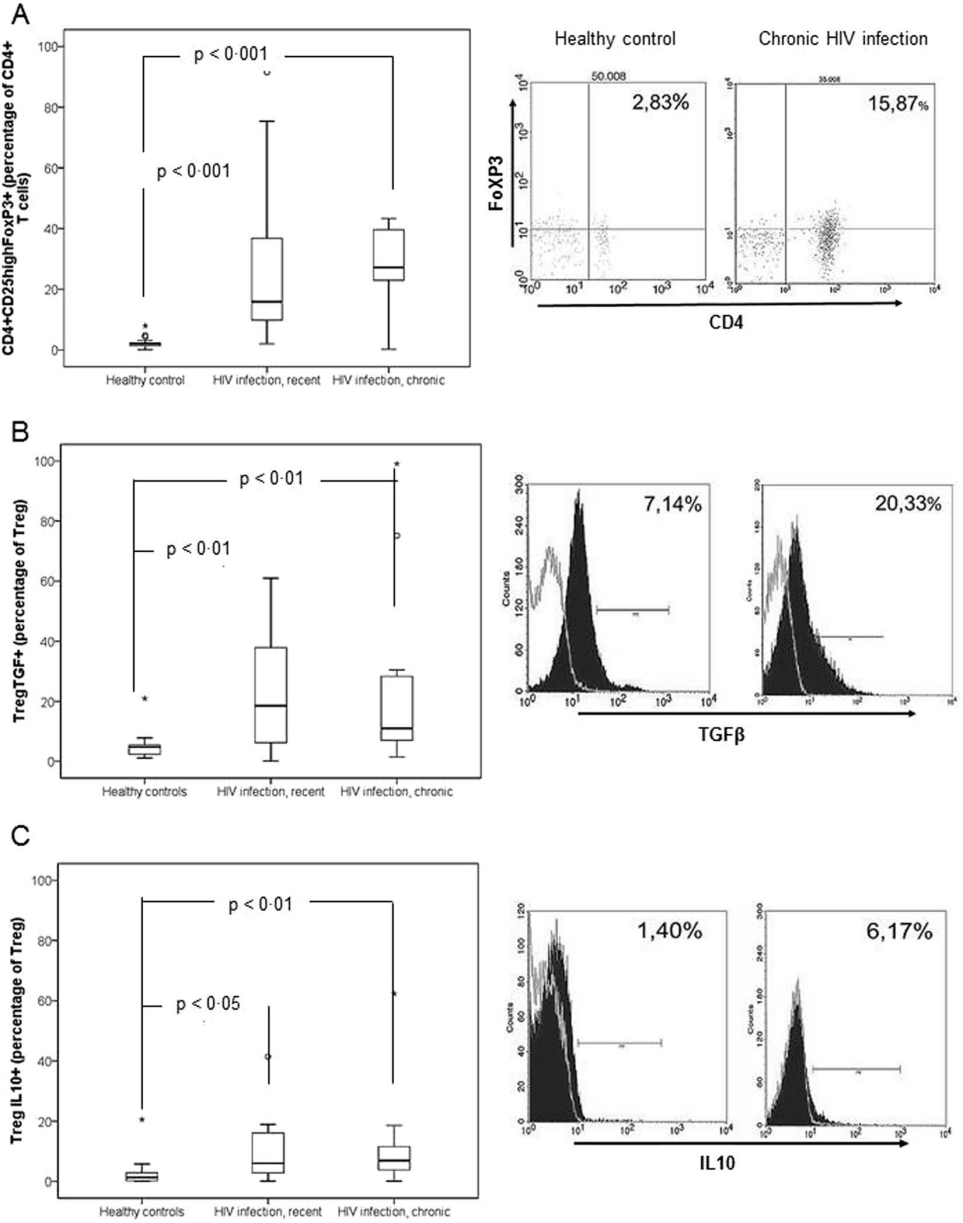
Proportions of T regulatory (CD4+ CD25highFoxP3+) cells and intracellular expression of cytokines by them in healthy controls (n = 20) and in untreated patients with HIV infection: recent (n = 10) and chronic (n = 10). (**A**) T regulatory cells (percentage of CD4+ T lymphocytes), determined after lysis and reaction with a mix of anti-CD3 (clone SK7), anti-CD4 (clone SK3), anti-CD25 (clone M-A251) and anti-FoxP3 (clone PCH-101) antibodies (BD Biosciences). (**B**) Intracellular expression of transforming growth factor beta 1 (percentage of T regulatory cells expressing transforming growth factor beta 1), determined by specific antibodies (anti-TGF-β1, clone BGh-LAP, BD Biosciences) and (**C**) interleukin 10 (percentage of T regulatory cells expressing interleukin 10), determined by specific antibodies (anti-IL-10,clone JES3-19F1, BD Biosciences), after PMA (30–50 ng/ml) and ionomycin (1 μg/ml) stimulation for 5 hours. For each graphic, flow cytometry data for representative cases of a healthy control and a patient with untreated chronic HIV infection are shown. A grey line in (**B**,**C**) figures represents isotype controls, used to confirm specificity of staining and to discriminate background staining. Data are provided as median, interquartile values and range.

Consistent with immune activation (detected by increased IL-6 and sCD14 concentration or percentages of CD4+ DR+ and CD8+ DR+), an increase in the numbers and percentages of regulatory cells and expression of immunoregulatory cytokines by them were expected. The absolute number and proportion of Tregs and the intracellular expression of TGF-β1 and IL-10 was significantly higher in HIV-infected individuals than in controls, without significant differences among the groups of HIV-infected patients (Table 3, Fig. 2).

**Table 3:** Number, percentage and expression of cytokines by regulatory cells of healthy controls and HIV-infected patients.

	Healthy controls (n = 20)	Naïve patients with detectable HIV load (n = 20).		
		Overall (n = 20)	HIV infection, recent (n = 10)	HIV infection, chronic (n = 10)
Treg/mm^3^	14 (9–24)	91 (19–258)**	113 (21–450)**	69 (18–255)*
Treg (percentage of CD4+ T cells)	2 (1–3)	23 (5–63)***	16 (6–55)***	27 (13–40)***
Treg expressing TGF-β1 (percentage of Treg)	5 (2–6)	16 (6–36)***	19 (5–42)**	11 (6–30)**
Treg expressing IL-10 (percentage of Treg)	1 (0–3)	6 (3–16)***	6 (3–18)*	7 (4–15)**
MDSC/mm^3^	13 (2–42)	13 (3–106)	16 (13–319)	6 (1–43)
MDSC (percentage of leukocytes)	0·3 (0·0–0·5)	0·3 (0·1–2·9)	0·5 (0·2–4·2)	0·1 (0·0–0·9)
MSDC expressing IL-10 (percentage of MSDC)	1 (1–2)	6 (4–13)***	5 (4–8)***	7 (4–22)***

Abbreviations: Treg: T regulatory cells (CD4+ CD25highFoxP3+); TGF-β1: Transforming growth factor beta 1; IL-10: interleukin 10; MSDC: Myeloid-derived suppressor cells [(CD3,CD14,CD19,CD56)neg, HLA-DRneg, CD11b+ and CD33+)].

Data are provided as absolute number (percentage) or as median (interquartile range).

HIV-infected patients vs healthy controls: *p < 0·05, **p < 0·01, ***p < 0·001.

Patients with recent vs chronic infection: ^‡^p < 0·05, ^‡‡^p < 0·01, ^‡‡‡^p < 0·001.

Among HIV-infected subjects, positive correlations between the proportions of Treg with CD4+ DR+ (r = 0·275, p = 0·040) and CD8+ DR+ (r = 0.289, p = 0·028) cells were observed. In HIV-infected individuals with recent or chronic infection and detectable HIV load, the percentage of Tregs correlated positively with the HIV load (r = 0·610, p = 0·006). However, the percentages of Tregs expressing TGF-β1 or IL-10 were not significantly correlated with the proportion of activated T cells or with the HIV load. In these untreated HIV-infected patients, the proportion of Tregs expressing TGF-β1 correlated negatively with the expression of IL-12 by mDCs (r = −0·489, p = 0·021) and IFN-α by pDCs (r = −0·514, p = 0·017). We did not observe any correlations between the percentages of Tregs and those of mDCs or pDCs.

**Figure 3:**
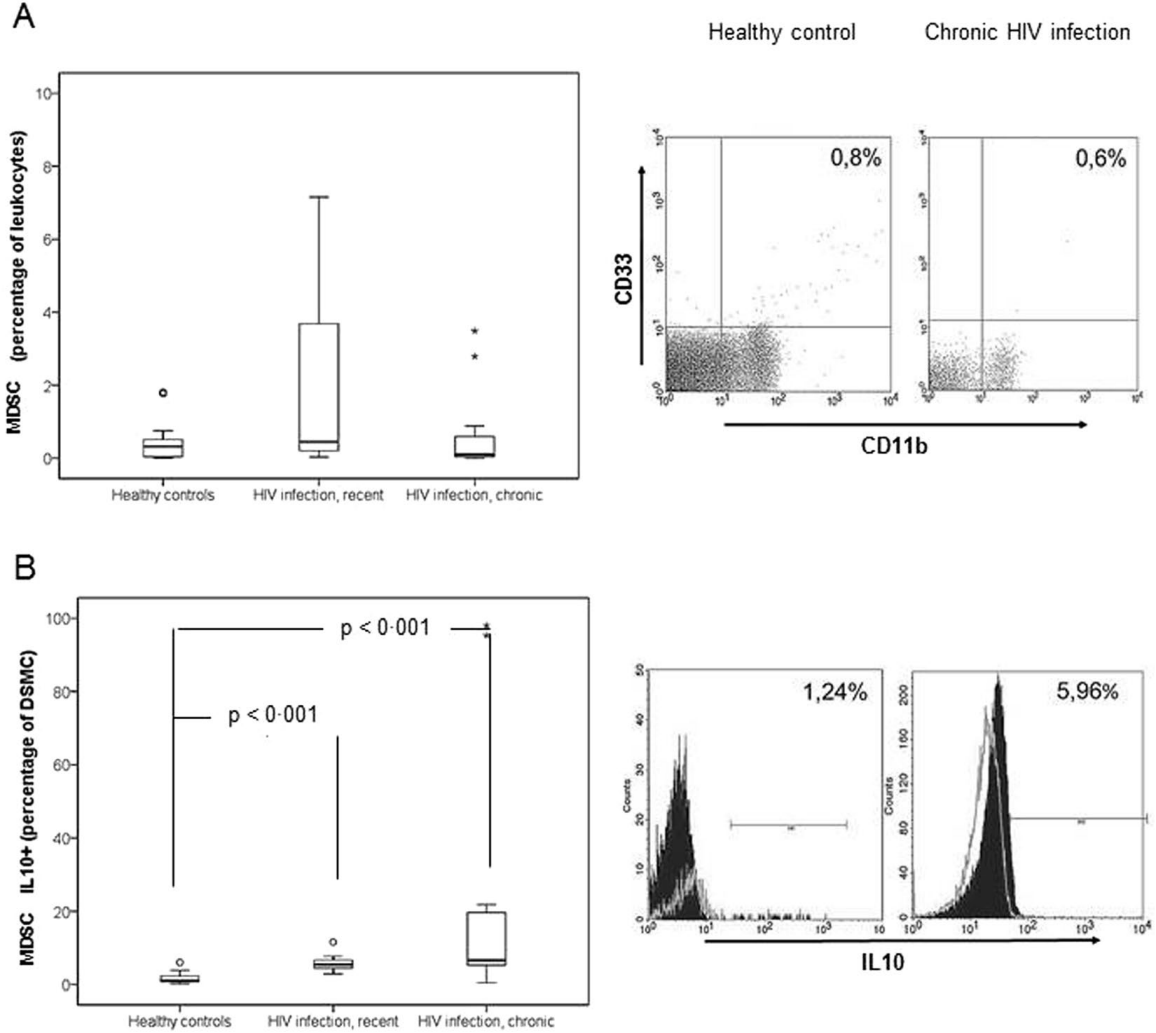
Proportions of myeloid-derived suppressor cells (MDSCs) and intracellular expression of interleukin 10 by them in healthy controls (n = 20) and in untreated patients with HIV infection: recent (n = 10) and chronic (n = 10). (**A**) MDSCs (percentage of leukocytes), quantified using anti-lineage (Cocktail Lin clones CD3-SK3, CD14-Mφ9, CD16-3G8, CD19-SJ25L1, CD20-L27,CD56-NCAM16.2.), anti-HLA-DR, anti-CD11b and anti-CD33 antibodies (BD Biosciences). (**B**) Intracellular expression of interleukin 10 (percentage of MDSCs expressing interleukin 10), determined using specific antibodies (anti-IL-10, clone JES3-19F1, BD Biosciences) after LPS (1 μg/ml) (Sigma) stimulation for 5 hours. For each graphic, flow cytometry data for representative cases of a healthy control and a patient with untreated chronic HIV infection with undetectable HIV load are shown. A grey line in (**B**) figure represents isotype controls, used to confirm specificity of staining and to discriminate background staining. Data are provided as median, interquartile values and range.

Next, the proportions of MDSCs were analyzed. No significant differences in the number or proportion of MDSCs between healthy subjects and HIV patients were detected. The intracellular expression of IL-10 was significantly higher in HIV patients but did not differ significantly among the groups of HIV-infected individuals (Table 3, Fig. 3). The percentage of MDSCs expressing IL-10 was not correlated with the percentages of activated lymphocytes, DC populations or the proportions of DCs expressing IL-12 or IFN-α.

### Evolution of bacterial translocation and immune markers after ART in patients with HIV infection and detectable viral load previously untreated

The evolution of immune populations after ART was different in patients with recent HIV infection compared with those with chronic HIV infection.

Nine of ten patients in each group started ART and achieved undetectable HIV load at 6 months. Recently infected patients were treated with tenofovir disoproxil, emtricitabine and darunavir/cobicistat (five individuals) or tenofovir disoproxil, emtricitabine and dolutegravir (four individuals). Chronically infected patients were treated with tenofovir disoproxyl, emtricitabine and darunavir/cobicistat (four patients), tenofovir disoproxil, emtricitabine and dolutegravir (one patient) or abacavir, lamivudine and dolutegravir (four patients). A significant increase in the CD4+ T cell count was observed in both groups of patients [Recent HIV infection: baseline, 572 (449–824)/mm^3^; after 12 month of ART, 769 (516 – 1278)/mm^3^, p = 0·039. Chronic HIV infection: baseline, 199 (132–340)/mm^3^; after 12 month of ART, 548 (361–924)/mm^3^, p = 0·002]. 16S rDNA was detected after 6 and 12 months of ART in all patients. Immune activation parameters (IL-6 and sCD14 concentrations and CD4+ DR+ and CD8+ DR+ cells) did not change significantly during follow-up (data not shown).

**Figure 4:**
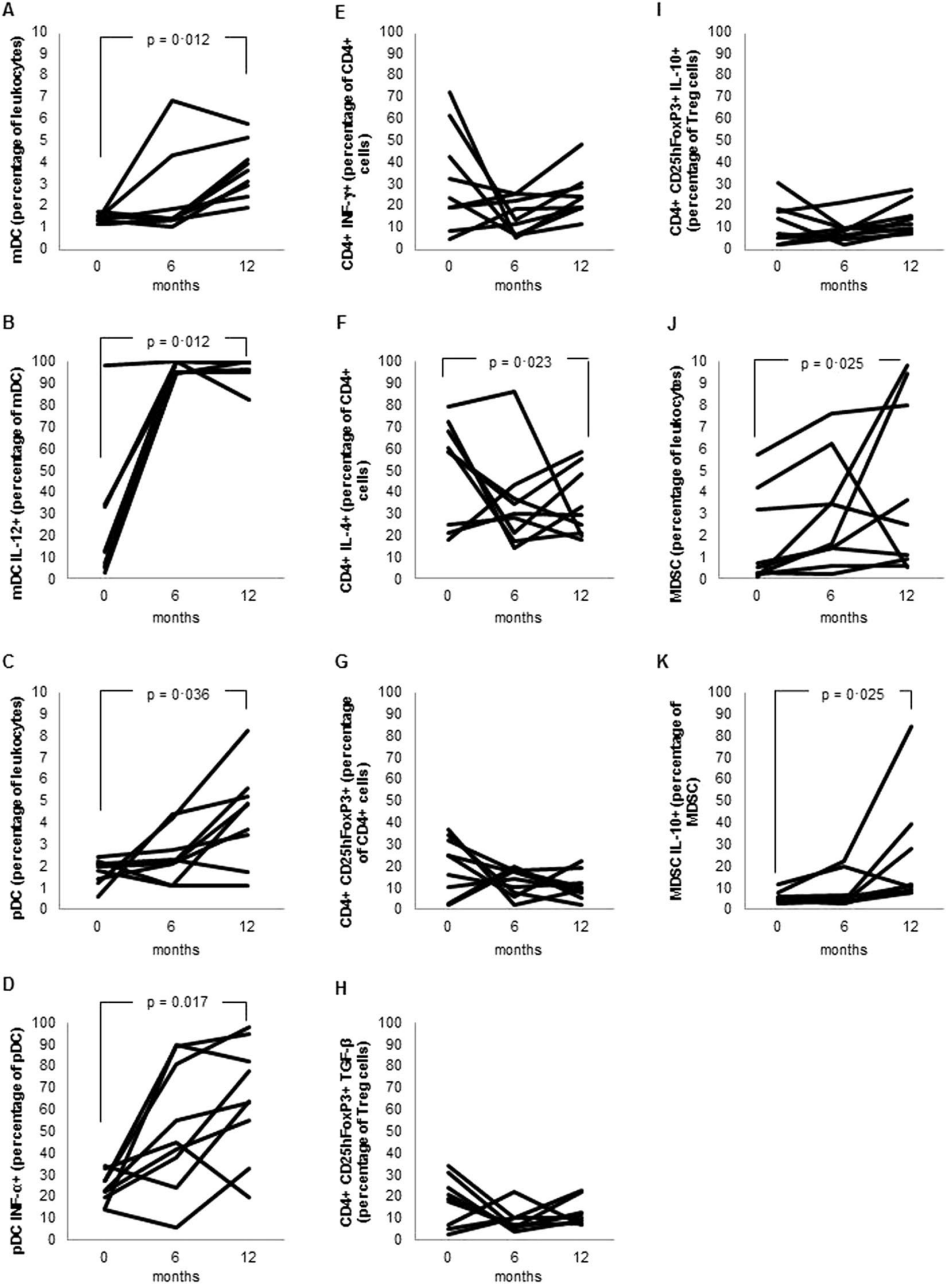
Evolution of cell populations in patients with recent (less than 6 months) HIV infection (n = 9) after 6 and 12 months of antiretroviral therapy. (**A**) Myeloid dendritic cells (percentage of leukocytes). (**B**) Myeloid dendritic cells expressing interleukin 12 (percentage of total myeloid dendritic cells). (**C**) Plasmacytoid dendritic cells (percentage of leukocytes). (**D**) Plasmacytoid dendritic cells expressing interferon alpha (percentage of total plasmacytoid dendritic cells). (**E**) Th1 cells (percentage of CD4+ T cells with intracellular expression of interferon gamma). (**F**) Th2 cells (percentage of CD4+ T cells with intracellular expression of interleukin 4). (**G**) T regulatory cells (CD4+ CD25highFoxP3+) (percentage of total CD4+ T cells). (**H**) T regulatory cells with intracellular expression of transforming growth factor beta 1 (percentage of T regulatory cells). (**I**) T regulatory cells with intracellular expression of interleukin 10 (percentage of T regulatory cells). (**J**) Myeloid-derived suppressor cells (percentage of leukocytes). (**K**) Myeloid-derived suppressor cells with intracellular expression of interleukin 10 (percentage of granulocyte myeloid-derived suppressor cells).

In patients with recent HIV infection, the analysis performed at the end of follow-up after ART revealed that the proportions of mDC and pDC had increased significantly as compared with the baseline values. Additionally, the intracellular expression of IL-12 and IFN-α by mDCs and pDCs, respectively, increased significantly after ART. The proportion of Th1 cells did not change significantly during follow-up, whereas the percentage of Th2 cells significantly decreased after ART. Whereas there was no significant change in the proportion of Tregs or in its expression of TGF-β1 or IL-10, significant increases in the proportion of MDSCs and the intracellular expression of IL-10 after ART were observed (Fig. 4).

**Figure 5:**
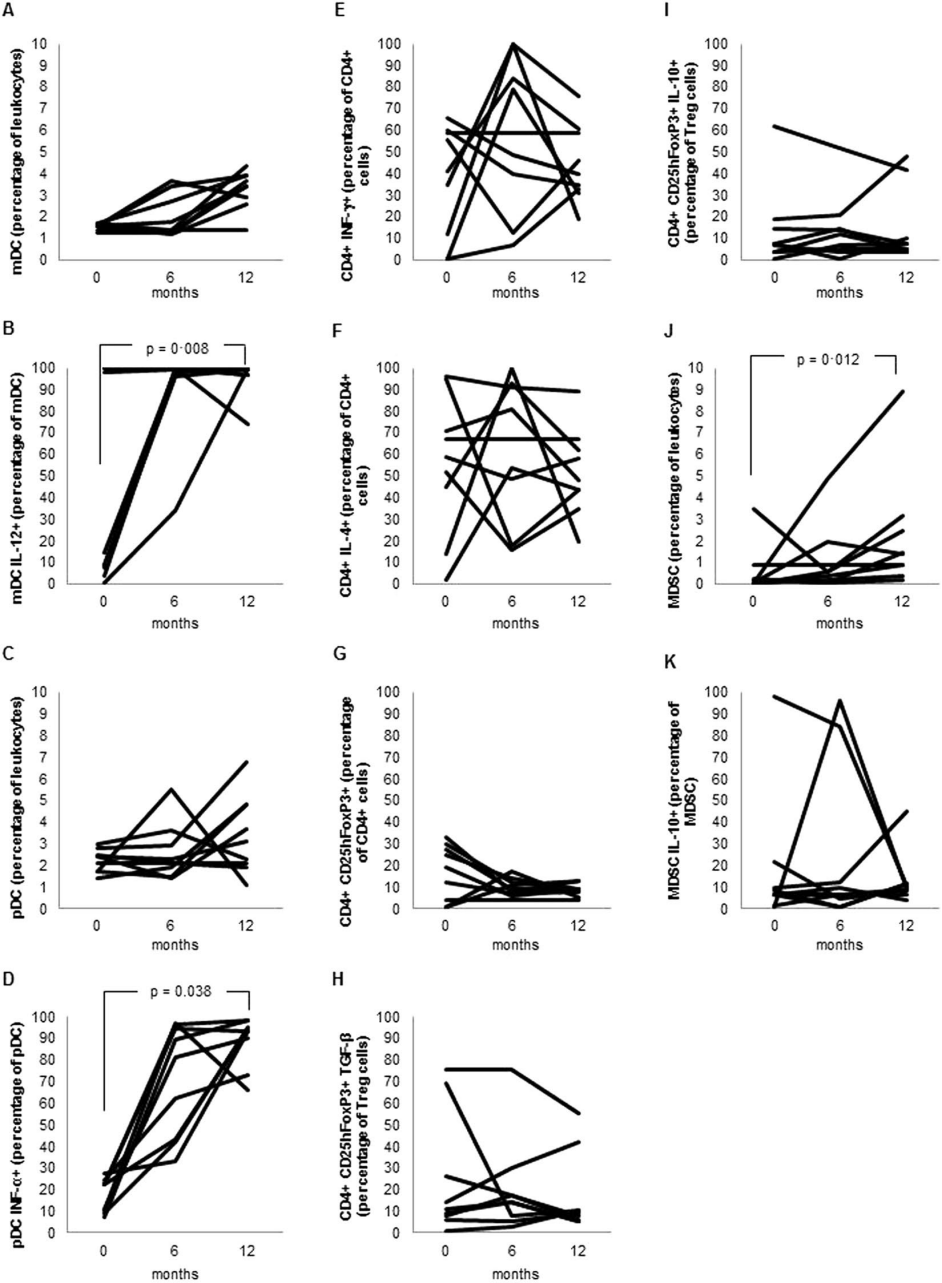
Evolution of cell populations in patients with chronic (more than 6 months) HIV infection (n = 9) after after 6 and 12 months of antiretroviral therapy. (**A**) Myeloid dendritic cells (percentage of leukocytes). (**B**) Myeloid dendritic cells expressing interleukin 12 (percentage of total myeloid dendritic cells). (**C**) Plasmacytoid dendritic cells (percentage of leukocytes). (**D**) Plasmacytoid dendritic cells expressing interferon alpha (percentage of total plasmacytoid dendritic cells). (**E**) Th1 cells (percentage of CD4+ T cells with intracellular expression of interferon gamma). (**F**) Th2 cells (percentage of CD4+ T cells with intracellular expression of interleukin 4). (**G**) T regulatory cells (CD4+ CD25highFoxP3+) (percentage of total CD4+ T cells). (**H**) T regulatory cells with intracellular expression of transforming growth factor beta 1 (percentage of T regulatory cells). (**I**) T regulatory cells with intracellular expression of interleukin 10 (percentage of T regulatory cells). (**J**) Myeloid-derived suppressor cells (percentage of leukocytes). (**K**) Myeloid-derived suppressor cells with intracellular expression of interleukin 10 (percentage of granulocyte myeloid-derived suppressor cells).

In patients with chronic HIV infection, no significant changes in the proportions of mDCs and pDCs were observed after 6 and 12 months on ART. However, the expression of IL-12 and IFN-α by mDCs and pDCs increased significantly at 6 and 12 months. After ART, we did not observe any significant differences in the percentage of Tregs or in TGF-β1 and IL-10 expression compared with the baseline values. A significant increase in MDSCs was detected in these patients after treatment, without significant modifications of IL-10 expression (Fig. 5).

Characteristics observed after 12 months of ART in these nine chronic HIV-infected individuals, untreated at baseline, were compared with those of 25 chronic infected patients with undetectable HIV at inclusion after a median of 54 (range, 16–97) months of ART [Age, 48 (39–53) years, Male sex 16 (64%), CD4/mm^3^ at diagnosis 185 (59–402), CD4/mm^3^ at inclusion 551 (403–743)]. No significant difference in bacterial translocation parameters (presence of 16S rDNA or LBP concentration) or in serum IL-6 or sCD14 levels between groups were observed. The absolute number and percentage of both mDC and pDC were significantly lower in patients with chronically controlled HIV load when compared with those controlled after only 12 months of ART. Likewise, significant differences were detected in the percentages of mDC expressing IL-12 and pDC expressing IFN-α between both groups. Th1 and Th2 cells number were similar in both groups. Chronically infected patients with undetectable HIV load at baseline exhibited significantly higher proportion of Treg expressing TGF-β1 and IL-10 than those controlled after only 12 months of ART (p < 0·05 in each case). The proportion of MDSCs and MDSCs expressing intracellular IL-10 were significantly lower in patients with chronically controlled HIV load (Table 4).

**Table 4:** Bacterial translocation parameters and immune characteristics of patients with chronic infection, untreated at baseline, analyzed after 12 months of antiretroviral therapy, and of chronically treated HIV patients with undetectable HIV load at baseline.

	Recent HIV-infected patients with detectable HIV load at baseline, after 12 months of antiretroviral therapy (n = 9)	Chronic HIV-infected patients with detectable HIV load at baseline, after 12 months of antiretroviral therapy (n = 9)	Chronic HIV-infected patients with controlled HIV load at baseline (n = 25)
Presence of bacterial DNA in peripheral blood (n, %)	9 (100)	9 (100)	25 (100)
LBP (ng/ml)	4·9 (3·8–6·6)	5·8 (5·5–8·3)	6·0 (4·9–7·1)
IL-6 (pg/ml)	4·5 (4·1–6·2)	5·7 (5·2–5·8)	5·4 (4·8–8·4)
sCD14 (ng/ml)	2142 (1942–2956)	3582 (2607–3694)	2902 (2446–3344)
T CD4+ DR+ cells (% of CD4+ lymphocytes)	10 (6–12)	13 (9–22)	13 (9–17)
T CD8+ DR+ cells (% of CD4+ lymphocytes)	11 (7–13)	16 (8–32)	12 (9–17)
mDC/mm^3^	168 (112–340)	95 (65–125)*	92 (76–143)^‡^
mDC (percentage of leukocytes)	3·2 (2·4–4·2)	3·6 (2·3–4·4)	1·7 (1·5–1·8)^‡‡‡, §§§^
mDC expressing IL-12 (percentage of mDC)	99 (95–100)	85 (26–99)	1·1 (0·6 – 1·8)^‡‡‡, §§^
pDC/mm^3^	223 (168–387)	102 (70–109)**	132 (107–154)^‡^
pDC (percentage of leukocytes)	4·8 (3·4–5·6)	3·9 (2·0–6·0)	2·2 (2·1–2·4)^‡‡, §^
pDC expressing IFN-α (percentage of mDC)	64 (33–95)	83 (50–95)	2 (1–2)^‡‡, §§^
Th1/mm^3^	162 (130–878)	300 (75–323)	252 (125–432)
Th1 (percentage of CD4+ T cells)	24 (20–69)	52 (30–65)	47 (32–63)
Th2/mm^3^	250 (131–699)	340 (88–368)	361 (139–473)
Th2 (percentage of CD4+ T cells)	33 (20–55)	53 (32–72)	54 (34–77)
Treg/mm^3^	79 (56–107)	68 (12–81)	57 (49–94)
Treg (percentage of CD4+ T cells)	9 (8–19)	8 (4–13)	10 (4–22)
Treg expressing TGF-β1 (percentage of Treg)	12 (9–23)	8 (5–51)	77 (44–92)^‡‡, §§^
Treg expressing IL-10 (percentage of Treg)	15 (10–25)	10 (5–52)	53 (47–61)^‡, §^
MSDC/mm^3^	2 (1–11)	5 (1–13)	3 (1–6)
MSDC (percentage of leukocytes)	11·7 (8·0–39·6)	5·2 (0·7–14·6)	0·3 (0·1–1·0)^‡‡, §^
MSDC expressing IL-10 (percentage of MSDC)	56 (7–74)	10 (6–54)*	6 (4–8)^‡‡, §^

Abbreviations: LBP: Lipopolysaccharide-binding protein; IL-6: interleukin 6; mDC: Myeloid dendritic cells; pDC: Plasmocytoid dendritic cells; IL-12: interleukin 12; IFN-α: interpheron alpha. Th1: T helper 1. Th2: T helper 2. Treg: T regulatory cells; TGF-β1: Transforming growth factor beta 1; IL-10: interleukin 10; MSDC: Myeloid-derived suppressor cells.

Data are provided as absolute number (percentage) or as median (interquartile range).

Chronic HIV-infected patients, detectable HIV load at baseline vs recent HIV-infected: *p < 0·05, **p < 0·01, ***p < 0·001.

Chronic HIV-infected patients, detectable HIV load at baseline vs recent HIV-infected: ^‡^p < 0·05, ^‡‡^p < 0·01, ^‡‡‡^p < 0·001.

Chronic HIV-infected, undetectable HIV load at baseline vs chronic HIV-infected patients, detectable HIV load at baseline: ^§^p < 0·05, ^§§^p < 0·01, ^§§§^p < 0·001.

## Discussion

In the present work, we have analyzed the characteristics of cells implicated in antigen presentation and down-regulation in HIV-infected patients before and after the initiation of ART. We have hypothesized that the changes in number or proportions of immune cells detected at baseline were due to the combined effect of HIV itself and microbial translocation-derived products. After control of HIV replication by ART, these microbial translocation-derived molecules would be the unique modulators of immune populations.

As it has previously communicated^11,12,14,15^, microbial translocation (as assessed by the presence of 16S rDNA or by the LBP levels in the serum), and monocyte (measured by IL-6 or sCD14 levels) and T lymphocyte (CD4+ DR+ and CD8+ DR+ percentages) activation were detected in recently and chronically infected patients, regardless of HIV load. Consistent with previous data^14,15^, we observed that microbial translocation, monocyte and lymphocyte activation persist after ART.

Infectious antigens are initially recognized by specialized antigen-presenting cells, including DCs. In contrast to previously reported data^3–9^, we observed that the percentages of DCs and the intracellular expression of IL-12 and IFN-α by mDCs and pDCs respectively, were higher in HIV-infected patients than in healthy controls. Our findings are in concordance with the expected increase in number and function of DCs after an infectious stimulus^1,2,23–26^. In patients with recent HIV infection, but not in those with chronic disease, who achieved an undetectable HIV viral load after ART, an increase in the number of both subtypes of DCs was observed, even though markers of bacterial translocation, monocyte and lymphocyte activation persisted unchanged. The increase in the proportions of mDCs and pDCs after ART in HIV patients with recent, but not in those with chronic, infection could be attributed to two causes: (1) Enhanced production of mDCs and pDCs. (2) Decrease in the loss of peripheral blood DCs by diminution of apoptosis or migration to lymphoid tissues. Any such change might be more significant in recently infected patients. Also, it could be secondary to the influence of Tregs on DCs: Tregs suppress DCs function through contact mechanisms and by the secretion of cytokines (TGF-β1 or IL-10)^17^; in fact, in our study the proportion of Tregs expressing TGF-β1 correlated negatively with the expression of IL-12 by mDCs and INF-α by pDCs. In both, recently and chronically HIV-infected patients, increased intracellular expression of IFN-α by pDC and IL-12 by mDC after 12 months of ART was observed, suggesting that the persistence of bacterial translocation may stimulate DCs continuously either through LPS/TLR4 or 16S rDNA/TLR9 interactions.

DCs play a key role in the adaptive immune response. It has been demonstrated a decrease in Th1 cells and an increase in Th2 cells^27^, supporting our data.

Lymphocyte activation, measured by CD4+ DR+ and CD8+ DR+ percentages, was present in HIV-infected patients and did not modify after control of HIV replication.

Several cells counteract immune activation in healthy humans. As it has previously reported^17,19,28,29^, we observed an increase in the proportion of Tregs in HIV-infected patients. In addition, we observed an increased proportion of Treg with intracellular TGF-β1 and IL-10 in HIV-infected individuals, supporting the enhanced regulatory function of these cells. The percentage of Tregs was significantly correlated with HIV load and with T cell activation markers.

The other regulatory population considered in this work was MDSCs, a heterogeneous population of immature and progenitor myeloid cells from monocyte or granulocyte lineage. MDSCs are characterized by strong immunosuppressive ability^18^. In contrast to previous reports, we did not observe increased MDSCs number in HIV-infected patients. Quin *et al*. described an expansion of monocytic MSDCs but not the granulocyte subset (CD11b+ CD33+ CD14− CD15+) in individuals with HIV^22^. It has been reported that the CD4+ T cell count influences the proportion of MDSCs. In agreement with our results, Volbrecht *et al*. reported that the MDSC levels of HIV individuals with a CD4 cell count of 250–500 cells/mm^3^ were not significantly different from those observed in controls^21^. Interestingly, we observed that the intracellular expression of IL-10, a cytokine with immunosuppressive function, by MDSCs was significantly increased in HIV-infected individuals. These effects have not been reported previously.

The percentage of activated lymphocytes and Tregs and their intracellular expression of TGF-β1 and IL-10 did not change significantly after ART, probably due to they continue down-regulating the increased immune activation. In contrast, a significant elevation of the number of MDSCs in both recently and chronically infected patients was detected. The exposure of precursors (peripheral blood or bone marrow mononuclear cells) to LPS or to proinflammatory cytokines contributes to the expansion of MDSCs^19,20^; this was a late effect because the expansion of MDSCs was not evident until 12 months of therapy. An increase in the expression of IL-10 by MDSCs after ART, probably as a consequence of maintained immune activation, was only detected in recently HIV-infected patients, suggesting a permanent functional alteration of MDSCs in those in which ART was initiated in a later phase.

Taken together the data of DCs and MDSCs, these findings suggest that in the early phases of infection, the response to antigenic stimuli and regulation of the immune activation are more preserved. These favorable modifications support the immediate initiation of ART, such it has been clinically proved^30,31^.

To analyze the persistence of these immune changes, a sample of chronic HIV-infected patients with ART-induced HIV undetectability for a period of 54 months was included. Bacterial translocation and immune activation markers continued being detected; thus, it was not surprising the elevated expression of immunosuppressive Treg-derived TGF-β1 and IL-10 detected in the patients. The down-regulating effects of these cytokines on DCs number and function^17^ could justify the observed diminution of DCs as well their expression of IL-12 and IFN-α observed in the present work by patients with ART-induced HIV undetectability for a long period. In fact the decrease observed in the number and function of pDCs and mDCs is in line with those articles which report a decreased proportion of both or the ability to secrete IL-12 and IFN-α by them^3–5,9,16^. Data about MDSCs were unexpected: a decreased percentage of MDSCs and MDSCs expressing IL-10 were detected in these patients. Two putative explanations are suggested: (1) Our measures of activation stimuli were not enough sensitive. LBP concentration was similar in these patients and in those with lower time of undetectability as well as IL-6 level, another stimulus of MSDCs formation^19,20^. (2) Alternatively, it is possible to speculate that MDSCs function as a relatively time-limited response to immune activation, being the Tregs those who exert the down-regulation during a more prolonged period.

In conclusion, the new findings about the pathogenesis of HIV infection supported by our results are: (1) The percentages of DCs and the intracellular expression of IL-12 and IFN-α by mDCs and pDCs, respectively, were elevated in HIV-infected patients. In patients with recent HIV infection, but not in those with chronic disease, an increase in the number of both subtypes of DCs after 12 months of ART was observed. However, in previously treated patients for a median of 54 months with undetectable HIV load, both mDC and pDC and the expression of IL-12 and IFN-α were significantly lower when compared with those controlled after only 12 months of ART, suggesting a decrease of them after a prolonged period of viral load undetectability. (2) Correlated with T cell activation markers, increased Treg proportion with elevated intracellular TGF-β1 and IL-10 expression was detected. In previously treated patients with undetectable HIV load for a median of 54 months, a significantly higher expression of TGF-β1 and IL-10 than in those controlled after only 12 months of ART was observed, suggesting a continuously attempt to control the cellular activation. (3) Finally, it was observed a significant elevation of the number of MDSCs expressing IL-10 in both recently and chronically infected patients compared with healthy controls. Increased numbers of MDSCs were detected in both groups of patients after ART. However, an enhanced percentage of MDSCs expressing IL-10 after ART was only detected in recently HIV-infected patients. The proportion of MDSCs and MDSCs expressing IL-10 were significantly lower in patients with chronically controlled HIV load (median time of undetectability, 54 months). The differences observed in previous parameters between individuals with recent or chronic HIV infection support the importance of early ART.

## Patients and Methods

### Study design

We conducted a prospective, observational study of consecutive cases of HIV infection recruited from a cohort of HIV-infected patients followed up in an HIV outpatient clinic at a University Hospital.

After providing informed consent, 45 HIV-infected adults were recruited. Of them, 20 naïve patients, including 10 recently infected patients [time since infection, 5 months (IQR 2–6)] and 10 chronically infected patients [time since infection, 53 months (IQR 12–59)] were included and prospectively followed after initiation of therapy. A sample of 25 patients with chronic HIV infection and ART-induced undetectable HIV viral load at baseline [undetectable HIV load for a median of 54 months (range, 16–97 months)] was also included to compare the results obtained in naïve patients after 12 months of treatment and those of patients with a longer period of undetectability. Twenty age- and gender-matched healthy individuals were recruited as controls from voluntary hospital workers.

The exclusion criteria were as follows: (1) Patients with clinical data suggesting acute HIV infection; (2) active or other opportunistic infections [including viral hepatitis, *Pneumocytis jirovecii*, toxoplasmosis, tuberculosis, cytomegalovirus infections, etc] or neoplasms. The screening procedure of them was that accepted by the Spanish Group for AIDS Study guidelines (www.gesida.seimc.org); (3) active drug use (cocaine, heroin, amphetamines) or significant alcohol ingestion (greater than 50 g/day); (4) treatments that could have modified the determination of inflammation-related molecules or cells (pentoxifylline, anti-inflammatory or immunosuppressive drugs); (5) red blood cell or plasma transfusion in the month before inclusion; or 6) antibiotic treatment, which might modify the presence of intestinal flora and the presence of blood bacterial DNA.

Patients at risk of HIV infection are tested every 6 months. Recent HIV infection was diagnosed if anti-HIV antibodies were present at inclusion but absent in a determination performed 6 months previously. Chronic infection was diagnosed if anti-HIV antibodies were present in a determination performed at least 6 months previously; these patients had refused ART when they were diagnosed. Controlled HIV replication was considered when the HIV load was less than 50 copies/ml (Abbott RealTime HIV-1, Abbott Park, IL, USA).

Duration of HIV infection was designated as the first positive anti-HIV test.

Bacterial translocation is usually indicated by increased serum levels of bacterial LPS, LPS-binding globulin (LBP) or bacterial 16S ribosomal DNA (16S rDNA)^32^. As recommended previously^32^, the presence of 16S rDNA and serum concentrations of LBP were used in this work.

### Study schedule

The study protocol included the following patient information: (1) clinical history, nadir CD4+ T cell count and HIV load, previous ART and time with undetectable HIV load; (2) CD4+ T cell count and HIV load at inclusion; and (3) peripheral blood sampling at inclusion for analysis of bacterial translocation and immune parameters. In those patients with detectable HIV loads, ART was initiated according to the Spanish Group for AIDS Study guidelines [www.gesida.seimc.org]. Current ART was continued in those patients with an undetectable HIV load.

Patients with a detectable HIV load at inclusion were followed for 12 months. Immune parameters were analyzed every six months.

### Laboratory methods

Blood samples were collected in pyrogen-free heparinized tubes (Biofreeze, Costar, USA) at 8 am to minimize the influence of circadian rhythms. 16S rDNA was detected after initial extraction of DNA (QIAamp DNA Mini Kit; QIAgen, Hilden, Germany) and quantification by spectrophotometry (BioRad, Hercules, CA, USA), posterior amplification of the 16S rDNA region of *E. coli*, using the following primers: 16SR 5′-ACC-GCC-ACT-GCT-GCT-GGC-AC-3′ y 16SF 5′-AGA-GTT-TGA-TCA-TGG-CTG-AG-3′ (IDT, Coralville, Iowa, USA). Polymerase chain reaction (PCR) was performed in Rotor Gene 6000 (QIAgen). The size of the band was measured by agarose gel electrophoresis.

LBP was measured by an immunometric sandwich assay (Immulite LBP; DPC, Los Angeles, CA, USA). Serum concentrations of IL-6 and soluble CD14 (sCD14) were quantified using the Quantikine Human Immunoassay (R&D, Minneapolis, MN, USA).

Cell suspensions from fresh peripheral blood samples were stained first with blue-fluorescent reactive dye (Live/Dead Fixable Dead Cell Stain Kit; Invitrogen, Carlsbad, CA, USA) to exclude dead cells from analysis, then for surface markers, and subsequently for intracellular molecules following fixation and permeabilization with the Intra Stain (Dako, Denmark A/S). DCs were analyzed on peripheral blood mononuclear cells (PBMC) after exclusion of monocytes, B lymphocytes and dead cells. A cocktail of anti-CD14/anti-CD19/dead cells (Cocktail Lin clones CD3-SK3, CD14-Mφ9, CD16-3G8, CD19-SJ25L1, CD20-L27, CD56-NCAM16.2; BD Biosciences, Franklin Lakes, NJ, USA) was used. For separate mDCs and pDCs, anti-CD1c (BDCA-1, clone AD5-8E7) and anti-CD303 (BDCA-2, clone AC-144) monoclonal antibodies, respectively, were used (Miltenyi Biotec GMBH, Bergisch Gladbach, Germany). Intracellular production of IL-12 by mDCs was determined using specific antibodies (anti-IL12p40/70, clone C8.6, Miltenyi Biotec GMBH) after stimulation with LPS (1 μg/ml) (Sigma, St Louis, Mo, USA) for 5 hours. Intracellular production of IFN-α by pDCs was determined using specific antibodies (clone LT 27:295, Miltenyi Biotec GMBH) after stimulation with CpG-A (ODN 2216) (3 μM) (Invivogen, San Diego, CA, USA) for 20 hours.

For T lymphocytes characterization, anti-CD3 (clone SK7), anti-CD4 (clone SK3), and anti-CD8 (clone SK1) antibodies were used. Activation of CD4+ and CD8+ T cells was determined by the membrane expression of CD3 (clone SK7), CD4 (clone SK3), CD8 (clone SK1) and HLA-DR (clone L243) (BD Biosciences). The Th1 and Th2 CD4+ populations were identified as CD4+ T cells expressing intracellular IFN-γ (clone 4S.B3) or IL-4 (clone MP4-25D2) (Th1 and Th2 populations), respectively, after stimulation with phorbol myristate acetate (PMA) (30–50 ng/ml) (Sigma) and ionomycin (1 μg/ml) (Sigma) for 5 hours.

The Treg count was determined after lysis and reaction with a mix of anti-CD3 (clone SK7), anti-CD4 (clone SK3), anti-CD25 (clone M-A251) and anti-FoxP3 (clone PCH-101) antibodies. Treg were defined as CD3+ CD4+ CD25highFoxp3+ cells. Intracellular production of TGF-β1 (clone BGh-LAP) and IL-10 (clone JES3-19F1) was analyzed using specific antibodies (BD Biosciences) after PMA (30–50 ng/ml) and ionomycin (1 μg/ml) stimulation for 5 hours.

MDSCs [(CD3,CD14,CD19,CD56)neg, HLA-DRneg (clone L243), CD11b+ (clone ICRF44) and CD33+ (clone P67,6)] were quantified using anti-lineage (Cocktail Lin clones CD3-SK3, CD14-Mφ9, CD16-3G8, CD19-SJ25L1, CD20-L27,CD56-NCAM16.2.), anti-HLA-DR, anti-CD11b and anti-CD33 antibodies (BD Biosciences). Intracellular production of IL-10 (clone JES3-19F1) was determined using specific antibodies (BD Biosciences) after LPS (1 μg/ml) (Sigma) stimulation for 5 hours.

In each case, unstimulated cells served as controls for the analysis of the synthesis of intracellular cytokines. Mouse Isotype controls conjugates with PE, PerCP, FITC and APC were used to confirm specificity of staining and discriminate the background.

Stained cells were washed, acquired and analyzed in a FACSCalibur cytometer, using CellQuest software (BD Bioscience, San Jose, CA, USA) for the analysis. In each case, 100.000 cells were acquired. The threshold was 52 FSC-H in all samples, the default value established in the FACSCalibur cytometer. Lymphocytes were gated based on forward (FSC-H) and size (SSC-H) scatter for determination of CD4+ and CD8+ populations. For characterization of lymphocytes CD4+ and CD8+, at the lymphocyte gate, we select by dot plot CD3+ CD4+, and within that gate, by histogram those who are DR+. For characterization of Th1 and Th2 lymphocytes, at the lymphocyte gate, we select by dot plot CD3+ CD4+, and within that gate, by histogram those lymphocytes with intracellular IFN γ or IL4. For characterization of Treg lymphocytes, in the gate of lymphocytes, we select those lymphocytes that were CD4+ CD25+ high intensity; within this gate, those lymphocytes with intracellular FoxP3.

mDCs were gated based on Lin– and DR-, and inside of this population BDCA-1+ and CD11c+ were selected. pDCs were gated based on Lin– and DR-, and inside of this population BDCA-2+ and CD123+ were selected.

MDSCs were gated based on Lin– and DR- and inside of this population CD11b+ y CD33+ were selected.

### Statistical analysis

Data were expressed as the absolute number (percentage) or as the median (25–75 interquartile range (IQR)). Categorical variables were compared using the chi-square test or Fisher’s exact test when necessary. Quantitative variables from independent groups were compared using the Mann-Whitney U test or ANOVA. Paired analysis of variables was performed using the Wilcoxon’s rank test. A two-tailed p value < 0.05 was considered significant. Statistical analysis was performed using the SPSS 18.0 statistical software package (SPSS Inc., Chicago, IL, USA).

### Ethical aspects

This study was performed according to the Helsinki Declaration. The project was approved by the ethical research committee of Hospital Puerta del Mar (Cádiz, Spain). Written informed consent was obtained from each participant.

## Data Availability

All data generated or analyzed during this study are included in this published article.
